# Fermentation Blues: Analyzing the Microbiota of Traditional Indigo Vat Dyeing in Hunan, China

**DOI:** 10.1128/spectrum.01663-22

**Published:** 2022-06-16

**Authors:** Shan Li, Yuru Shi, Hui Huang, Yan Tong, Shaohua Wu, Yuhua Wang

**Affiliations:** a Key Laboratory for Microbial Resources of the Ministry of Education, Yunnan Institute of Microbiology, School of Life Sciences, Yunnan University, Kunming, China; b Department of Economic Plants and Biotechnology, Yunnan Key Laboratory for Wild Plant Resources, Kunming Institute of Botany, Chinese Academy of Sciences, Kunming, China; USDA – San Joaquin Valley Agricultural Sciences Center

**Keywords:** traditional indigo fermentation, specific plant mixture, indigo reduction, *Pseudomonas*, *Alkalibacterium*, fungal diversity

## Abstract

Traditional indigo dyeing through anaerobic fermentation has recently gained worldwide attention in efforts to address concerns regarding the sustainability of industrial indigo dyeing and the impact of toxic reducing agents such as sodium dithionite (Na_2_S_2_O_4_) on human health and the ecological environment. Intriguingly, changes in the microbiota during indigo fermentation are known to potently affect the onset of indigo reduction, and thus elucidation of the microbial community transitions could help develop methods to control the initiation of indigo reduction. Here, we investigated the microbiota associated with the traditional indigo dyeing practiced in Hunan, China. Specifically, we identified the bacterial and fungal components of the microbiota at distinct stages in the indigo fermentation process by analyzing 16S rRNA gene and internal transcribed spacer sequences. Our analyses revealed two substantial changes in the microbiota during the traditional indigo fermentation process. The first change, which was probably caused by the introduction of Chinese liquor (featuring a high alcohol concentration), resulted in decreased bacterial diversity and increased proportions of Pseudomonas, Stenotrophomonas, and Bacillaceae family members. The second change, which could be attributed to the addition of specific plant species, led to an increase in the abundance of Alkalibacterium, Amphibacillus, the obligate anaerobe Turicibacter, the facultative anaerobe Enterococcus, and *ZOR0006*, as well as to a decrease in the pH and redox potential values. Our results indicate that the specific plant mixture included in the procedure here could be used as an effective additive to accelerate the initiation of indigo reduction during the fermentation process. To the best of our knowledge, this is the first report revealing the fungal diversity during the indigo fermentation process and, furthermore, showing that the fungal diversity has remained in transition despite the relatively stable bacterial diversity in the proper indigo fermentation process. Although traditional indigo fermentation in China is challenging to manage, we can benefit from local knowledge of the fermentation process, and understanding the scientific bases of traditional indigo fermentation will facilitate the development of environmentally friendly procedures.

**IMPORTANCE** Chemical reducing agents included in modern indigo dyeing to initiate indigo reduction can be harmful to both human health and the environment. Given that traditional indigo dyeing involves natural fermentation in a dye vat using natural organic additives without the use of toxic chemicals and that changes in the microbiota during traditional indigo fermentation potently affect the onset of indigo reduction, elucidation of these microbial community transitions could help develop methods to control the initiation of indigo reduction. This study on the microbiota associated with the traditional indigo dyeing practiced in Hunan, China, has identified the bacterial and fungal communities at distinct stages of the indigo fermentation process. Notably, the addition of specific plant species might yield the desired microbial communities and appropriate fermentation conditions, which could be used as an effective additive to accelerate the initiation of indigo reduction. This study has also revealed the fungal diversity during the indigo fermentation process for the first time and shown that the fungal diversity has remained in transition despite the relatively stable bacterial diversity. Thus, this work provides new insights into the traditional indigo fermentation process used in China and substantially enhances current efforts devoted to designing environmentally friendly methods for industrial indigo dyeing.

## INTRODUCTION

Fermentation has long been used in the production of diverse fermented products, including rice wine, pickled vegetables, tea, and cheeses, and several of the fermentation processes involved are performed under microaerobic or anaerobic conditions. One of the oldest fermentation techniques is indigo fermentation, which reduces indigo pigment to a water-soluble form (leuco-indigo) through natural anaerobic fermentation for application in traditional indigo dyeing; archeological evidence has recently been obtained of indigo-dyed textiles from at least 6,000 years ago (1). Intriguingly, diverse recipes of indigo fermentation processes have been traditionally used in various parts of the world for vat dyeing, such as the medieval woad vat in Europe, *sukumo* vat in Japan, and sweet indigo vat in India, and many of these recipes have been studied or described (2). However, because of the uncertainty regarding the initiation of indigo reduction in the natural indigo fermentation process, chemical reducing agents such as sodium dithionite (Na_2_S_2_O_4_) must be included in the modern indigo dyeing (3). Consequently, elucidation of the mechanisms underlying the initiation of indigo reduction is one of the crucial challenges associated with natural indigo fermentation. Previous studies have addressed the diversity and dynamics of the bacterial community during the entire indigo fermentation process by using culture-independent and culture-dependent methods in Japan, Korea, and Europe (3–9). Most of these studies have shown that the initiation of indigo reduction is a complex process in which the most aerobic bacteria and Pseudomonas are gradually replaced by a microbial community dominated by Alkalibacterium, Amphibacillus, and the most facultative or obligate anaerobes (3–5, 7–9). The indigo-reduction reaction occurs due to the action of a group of bacteria collectively known as indigo-reducing bacteria, of which ~15 species have been identified and isolated from fermentation broths (10–23). The aforementioned studies attempted to identify the bacterial community involved in the indigo fermentation process, but the potential occurrence of fungi during the process was not reported. Furthermore, distinct forms of indigo dye, such as couched woad, *sukumo*, or indigo paste, have been used in the traditional dyeing process, and the microbiota in indigo fermentation has been widely shown to be highly influenced by the different forms of indigo dye and the fermentation methods used (3, 4, 24).

In contrast to other indigo fermentation processes, the traditional indigo fermentation process used in China has not been elucidated; the preparation procedure for this fermentation involves adding specific plants that are extremely distinct from other recipes, and the role of the plants added in the fermentation process also remains unclear. Moreover, the indigo paste used by the Dong people in China is produced using the indigo dye extracted from Strobilanthes cusia and Polygonum tinctorium (the origin of indigo in other traditional dyeing processes is either S. cusia or P. tinctorium, rather than mixing them). The indigo extraction process used by the Dong people is as follows: (i) fresh leaves and stems are harvested from the indigo-producing plants (S. cusia or P. tinctorium) and then soaked in the water for 2 to 3 days; (ii) the plant residues are removed when the stems and leaves are soft; (iii) lime is added to the liquid and then the liquid is stirred vigorously for about half an hour; (iv) after stirring is completed, the suspension is left alone for several hours; and (v) after the indigo paste settles to the bottom, the supernatant is discarded to finally obtain the indigo paste. Consequently, the microbiota associated with the traditional indigo fermentation method used by the Dong people in China could differ from that associated with the Japanese method employing *sukumo* or the European method employing couched woad.

Thus, we aimed to reveal the microbial species that occur during the traditional indigo fermentation process used by the Dong people in China, because this could provide new insights into the core microbial community transitions associated with the state of indigo reduction. Furthermore, understanding why specific plants are added in the dyeing vat is critical, and we hypothesize, for example, that the plants could accelerate the fermentation process.

## RESULTS

### Environmental parameters.

**FIG 1:**
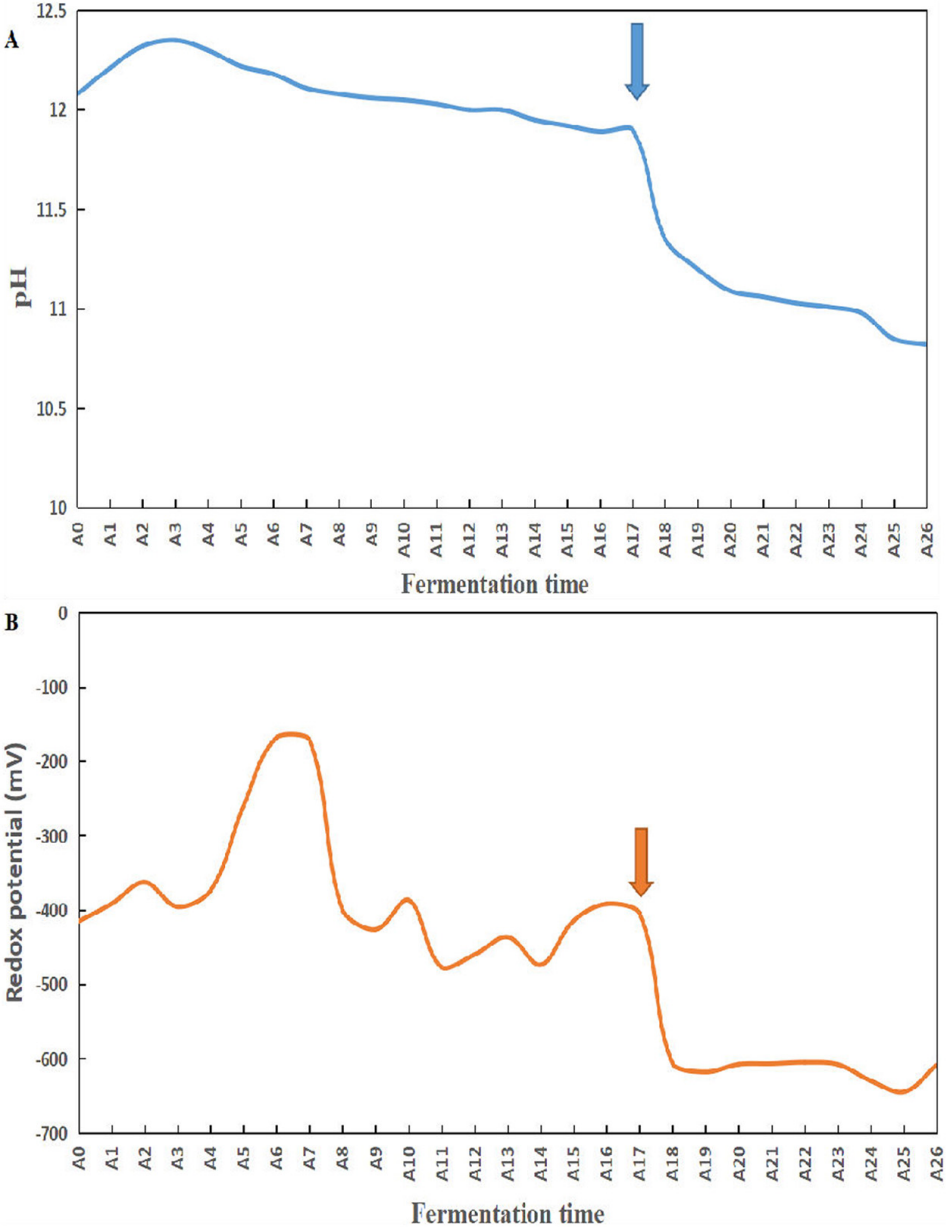
Evolution of pH (A) and redox potential (B) during the traditional indigo vat dyeing process. Arrows indicate time of addition of the specific plant species.

The changes in pH, redox potential, and temperature were monitored during the traditional indigo fermentation process. Fig. 1 shows the measured fluctuations in pH and redox potential. The changes of temperature are shown in Fig. S3. After specific plant species were added on the 18th day, the pH of the fermentation liquor dropped from 11.9 to 11.35, and the oxidation reduction potential (ORP) dropped from −404.5 to −606.8 mV. Moreover, the pH continued to decrease further until the last day of the fermentation, whereas the ORP remained stable until the 24th day, gradually decreased to −645 mV by the 26th day, and then increased to −608.7 mV by the last day.

### Sequencing analysis.

We obtained 3,488,336 and 4,205,676 raw reads from the sequencing of the16S rRNA gene (V3 to V4 region) and internal transcribed spacer (ITS) amplicon (ITS1 region), respectively. This resulted in 1,048,068 sequences, ranging from 25,066 to 60,285 per sample (*n* = 26), representing 4,098 ASVs in the case of the 16S rRNA gene data; and in 1,705,524 sequences, ranging from 45,663 to 96,344 per sample (*n* = 26) representing 3,361 ASVs in the case of the ITS data. In order to complete downstream diversity and composition analyses, sequences were rarefied to the lowest numbers, which were 12,492 and 45,663 sequences per sample for the 16S rRNA gene and ITS, respectively. This resulted in the amplicon sequences clustering into 3,659 and 3,342 ASVs for the 16S rRNA gene and ITS data, respectively.

### Microbial community composition.

**FIG 2:**
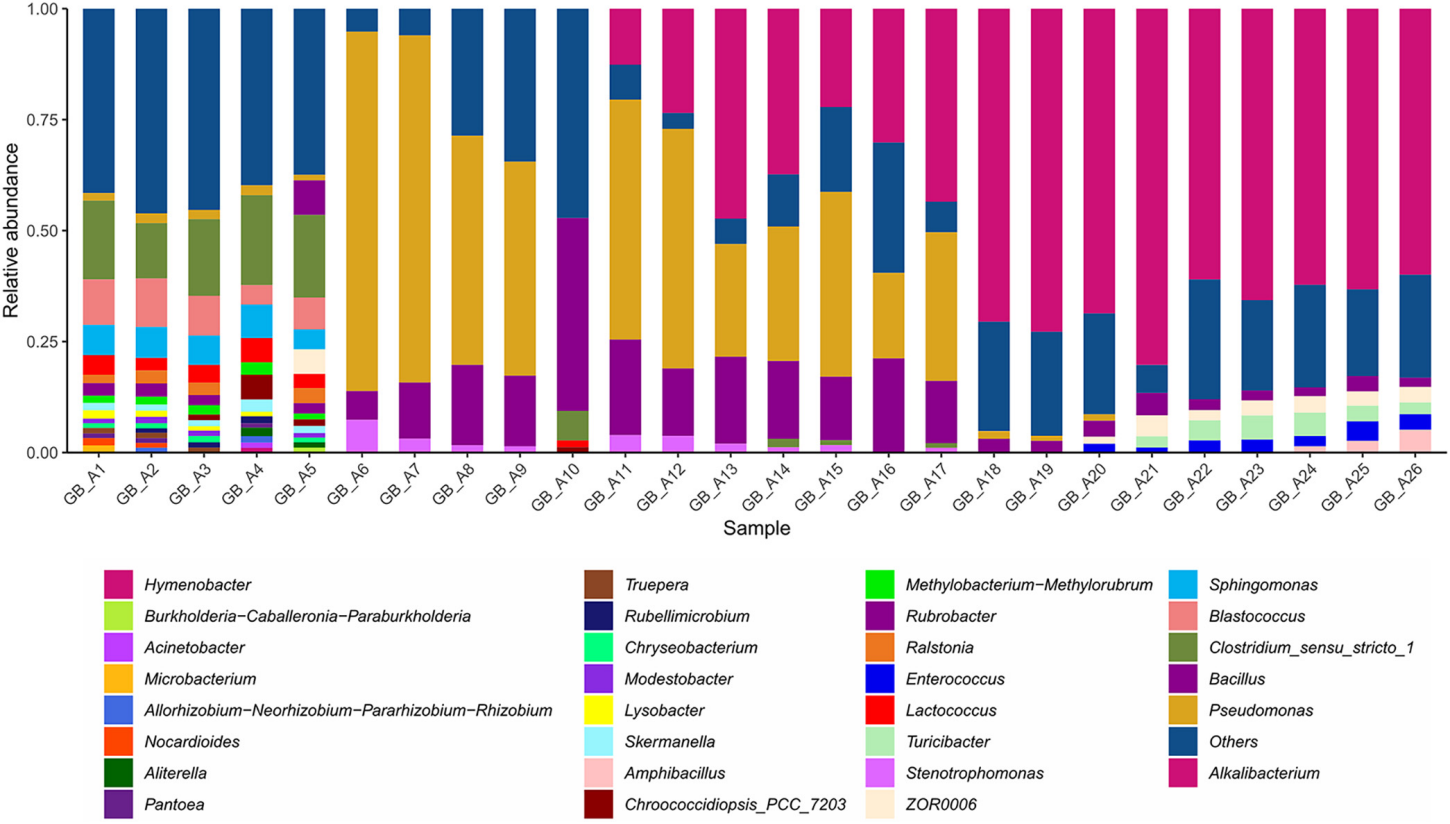
Relative abundance of bacterial genera in indigo fermentation fluids over time. Bacterial genera detected at a relative abundance of ≤1% are classified as “Others.”

To understand the succession of the microbial communities during the traditional indigo fermentation process, the fermentation fluid was collected from the 2nd day to the 27th day—from the initiation of fermentation until the initiation of reduction—and the samples were analyzed using next-generation sequencing. In the collected samples, 859 bacterial genera from 37 phyla were detected, and the bacterial community in the fermentation process was dominated at the phylum level by Firmicutes (52.1%), Proteobacteria (29.5%), and Actinobacteria (15.8%); the total relative abundance of the remaining 34 phyla combined was <3%. Moreover, 581 fungal genera from 10 phyla were detected in the samples, with the most abundant phyla being Ascomycota (67.3%) and Basidiomycota (19.6%). The fungal community composition during the fermentation process is shown in Fig. S4. Fig. 2 shows the temporal patterns of the bacterial community structure during the fermentation process. The number of bacterial species decreased dramatically after the fermentation liquid was treated with Chinese liquor, going from 398 species on day 2 to 117 species on day 7. Moreover, bacterial diversity was higher in the fluid from the early stage of fermentation (days 2 to 6), with an average of 390 species detected per sample; our sequence analysis revealed that the number of bacterial species at the beginning of fermentation was ~10-fold higher than that at the initiation of reduction (the dye liquid turned yellow-green in color and could be used to dye cloth). On the 7th day, the major constituents were Pseudomonas (81.0%), Stenotrophomonas (7.4%), and Bacillaceae family members (6.5%); these were the predominant taxa after the introduction of Chinese liquor and their dominance continued until the 18th day. On the 10th day, the proportion of the genus Alkalibacterium was negligible (0.02%), but Clostridium_sensu_stricto_1 appeared on the 11th day (6.6%) and was followed by an abrupt increase in the proportion of Alkalibacterium on the 12th day (12.6%). The number of bacterial species decreased from 106 on day 18 to 38 on day 19, which corresponded with the time at which the specific plant species were added to the fermentation liquor. Erysipelotrichaceae appeared on the 19th day (Fig. S1). After the plant species were added, Pseudomonas, Stenotrophomonas, and Bacillaceae family members tended to decrease in overall relative abundance or disappear completely. Considering the indigo-reducing bacteria that have been reported previously, the most likely indigo-reducing population here includes Amphibacillus and Alkalibacterium. Alkalibacterium increased in proportion from 43.5% on the 18th day to 70.5% on the 19th day and was the most numerous taxon thereafter. In the case of Amphibacillus, the proportion was 1.4% on day 25 and then 2.6% on day 26, and the relative abundance of this genus peaked on day 27 (5.1%). An upward trend was also observed with the proportions of the obligate anaerobe Turicibacter, the facultative anaerobe Enterococcus, and *ZOR0006*. The drastic changes measured in the microbiota coincided with the time at which selective pressure was applied through the introduction of the specific plant species in the fermentation. Furthermore, the relatively high differences in values between days 18 and 19 also aligned with the changes in pH and ORP.

**FIG 3:**
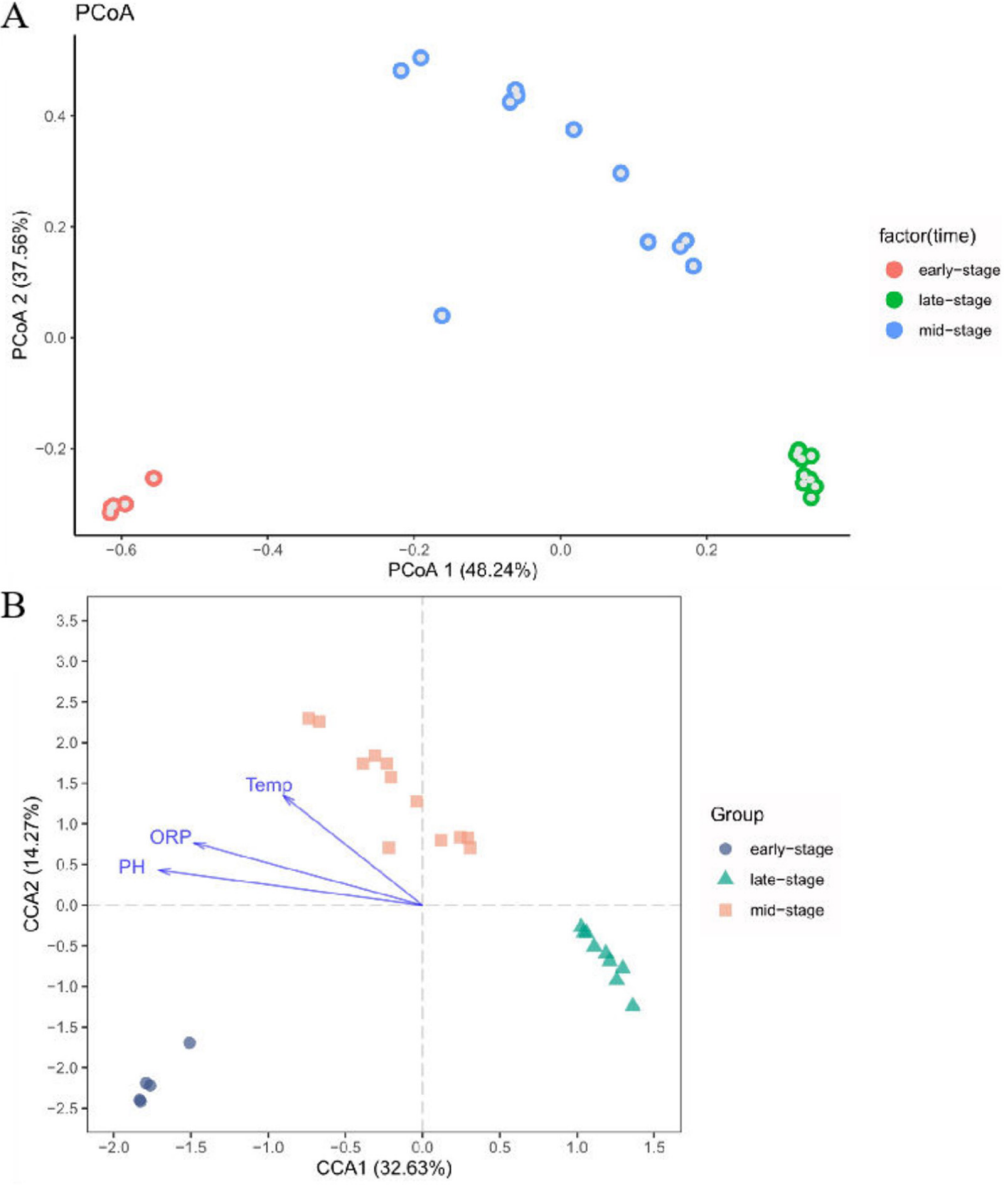
(A) Principal coordinate analysis (PCoA) plot of bacterial community changes during the fermentation process. Colored dots indicate bacterial microbiome in individual samples. (B) Canonical correlation analysis (CCA) of environmental variables and bacterial community structures at the genus level. ORP, oxidation reduction potential.

Next, principal coordinates analysis (PCoA) was used to compare the structure of the bacterial community in the indigo fermentation process. The results are presented in Fig. 3A, where the horizontal coordinate indicates one principal component, the vertical coordinate indicates another principal component, and the percentages indicate the respective contributions of each principal component to the sample variance. The entire fermentation process could be divided into three stages: early stage (days 2 to 6), middle stage (days 7 to 18), and late stage (days 19 to 27), based on Bray-Curtis diversity metrics by using the Anosim statistical test (*P = *0.001). Each point in the figure represents one sample, and samples at the same stage are indicated by the same color. Our results showed that the bacterial community structures differed significantly between the early, middle, and late stages. The microbiota changed slowly from the 2nd day to the 6th day, but after Chinese liquor was added on the 6th day, the bacterial composition changed rapidly. Subsequently, the bacterial composition changed continuously from the 7th day to the 18th day. Moreover, after addition of the plant species on the 18th day, the bacterial composition was considerably altered on the 19th day; however, the change in the microbiota from the 19th day to the 27th day was relatively small, which implied that the microbiota had become highly stable. These results indicated that adding Chinese liquor on the 6th day and the specific plant species on the 18th day potently influenced the composition of the bacterial community.

Previous studies have identified pH, redox potential, and temperature as critical environmental parameters during the fermentation process. Therefore, we performed canonical correlation analysis (CCA) to assess the potential relationships between these environmental parameters and the bacterial community composition (Fig. 3B): all three parameters (pH, ORP, and temperature) were significantly correlated with bacterial community composition (*P < *0.05). Our CCA results further confirmed that pH (*r*^2^ = 0.9336, *P = *0.001) and ORP (*r*^2^ = 0.815, *P = *0.001) play an important role in the bacterial community composition during the indigo fermentation process; pH, redox potential, and temperature were positively correlated with the bacterial community in samples from the early and middle stages (*P < *0.05) but negatively correlated with the bacterial community in the late-stage samples.

**FIG 4:**
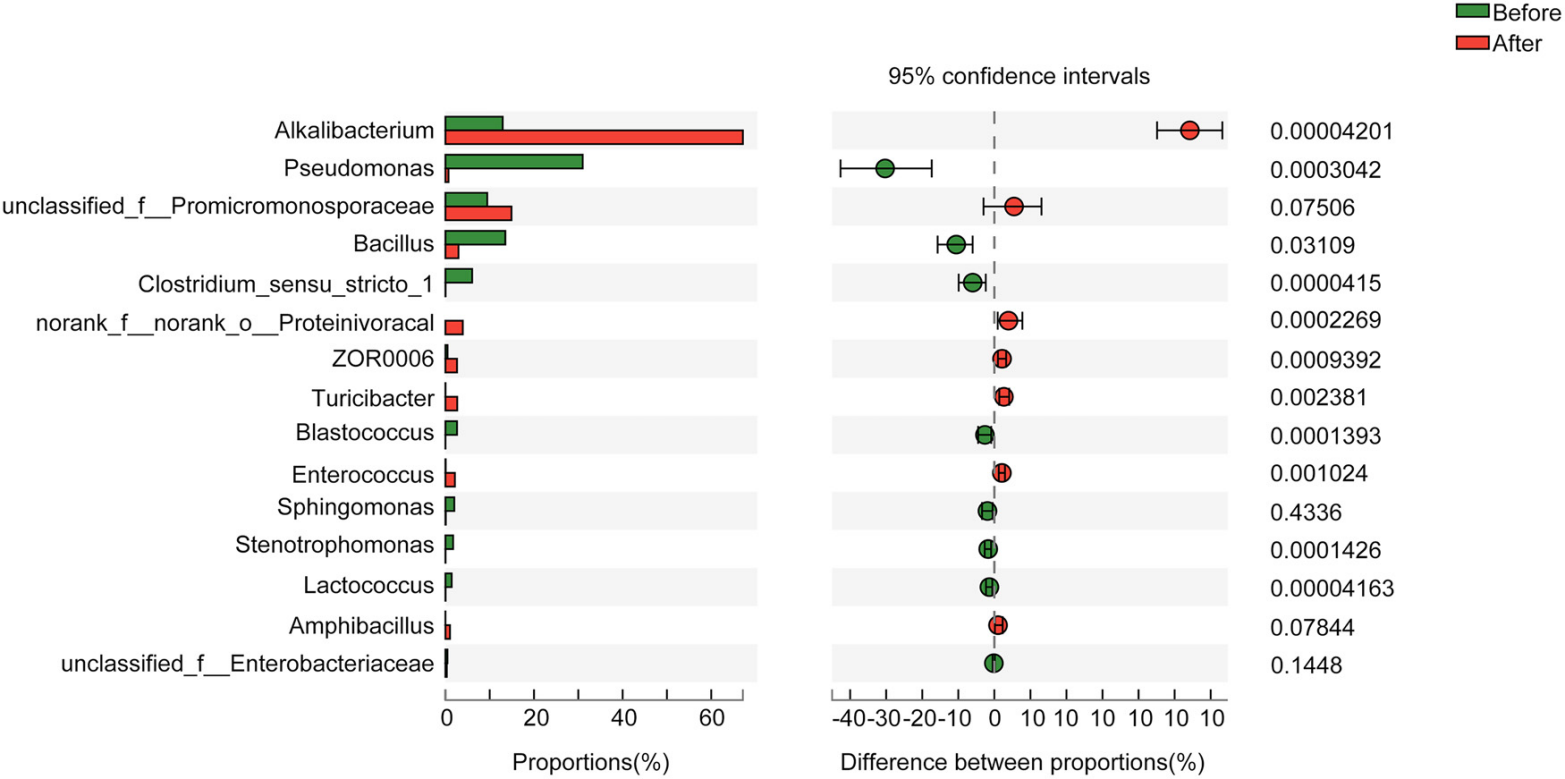
Graph showing that the relative abundances of bacterial genera in the indigo fermentation samples are related to the addition of the specific plant species.

Addition of the specific plant species on the 18th day strongly affected the bacterial richness and composition of the indigo fermentation samples. Thus, we further investigated and identified the taxa at the genus level before and after the addition of the plant species: whereas 161 genera overlapped between the two stages, 25 genera that were present at the end of fermentation (day 19 to day 27) were not detected before the plant species were added. Moreover, we performed a differential abundance analysis by using Wilcoxon tests to identify the taxa whose abundance differed significantly between the two groups (Fig. 4): before the specific plant species were added, Pseudomonas, Bacillus, and Clostridium_sensu_stricto_1 were the genera that were significantly more abundant; conversely, after the plant species were added, the proportion of Alkalibacterium increased significantly.

### Correlations between the bacteria and fungi in indigo fermentation process.

**FIG 5:**
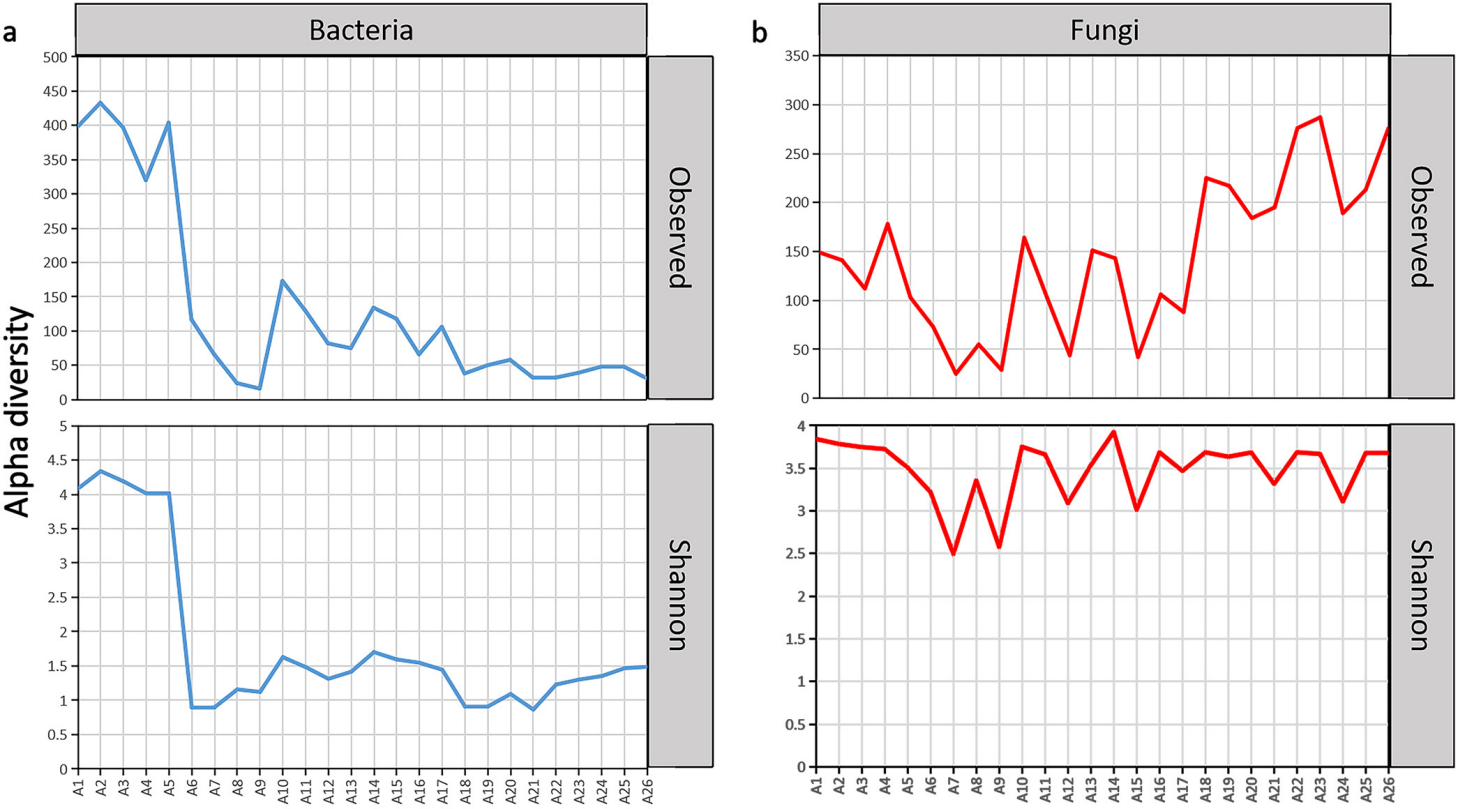
α diversity changes of bacteria (a) (16S rRNA gene) and fungi (b) (internal transcribed spacer [ITS]) in indigo fermentation samples.

The first 6 days of the traditional fermentation process is failed to initiate indigo reduction, so that the correlation at this stage is not relevant to a proper indigo fermentation process. We did not observe any significant correlations in α diversity based on Spearman correlations (from days 7 to 27). Changes in α diversity of bacteria and fungi (based on observed richness and Shannon diversity index values) depending on the fermentation time are shown in Fig. 5. The fungal diversity has remained in transition despite the relatively stable bacterial diversity in the proper indigo fermentation process (except the first 6 days).

We further assessed the correlations both within and between the fungal and bacterial microbiomes for the 35 most abundant genera. Most of the bacterial microbiota showed significant positive correlations (*P < *0.05) (Fig. S2). Significant negative correlation was observed between Alkalibacterium and Ralstonia (*r* = −0.72), Amphibacillus and Bacillus (*r* = −0.54), and Turicibacter and Pseudomonas (*r* = −0.72), Stenotrophomonas (*r* = −0.68), and Bacillus (*r* = −0.62), whereas positive correlation was observed between Turicibacter and Enterococcus (*r* = 0.77). Unclassified_f_Enterobacteriaceae was negatively correlated with Pseudomonas (*r* = −0.60) and Stenotrophomonas (*r* = −0.57).

**FIG 6:**
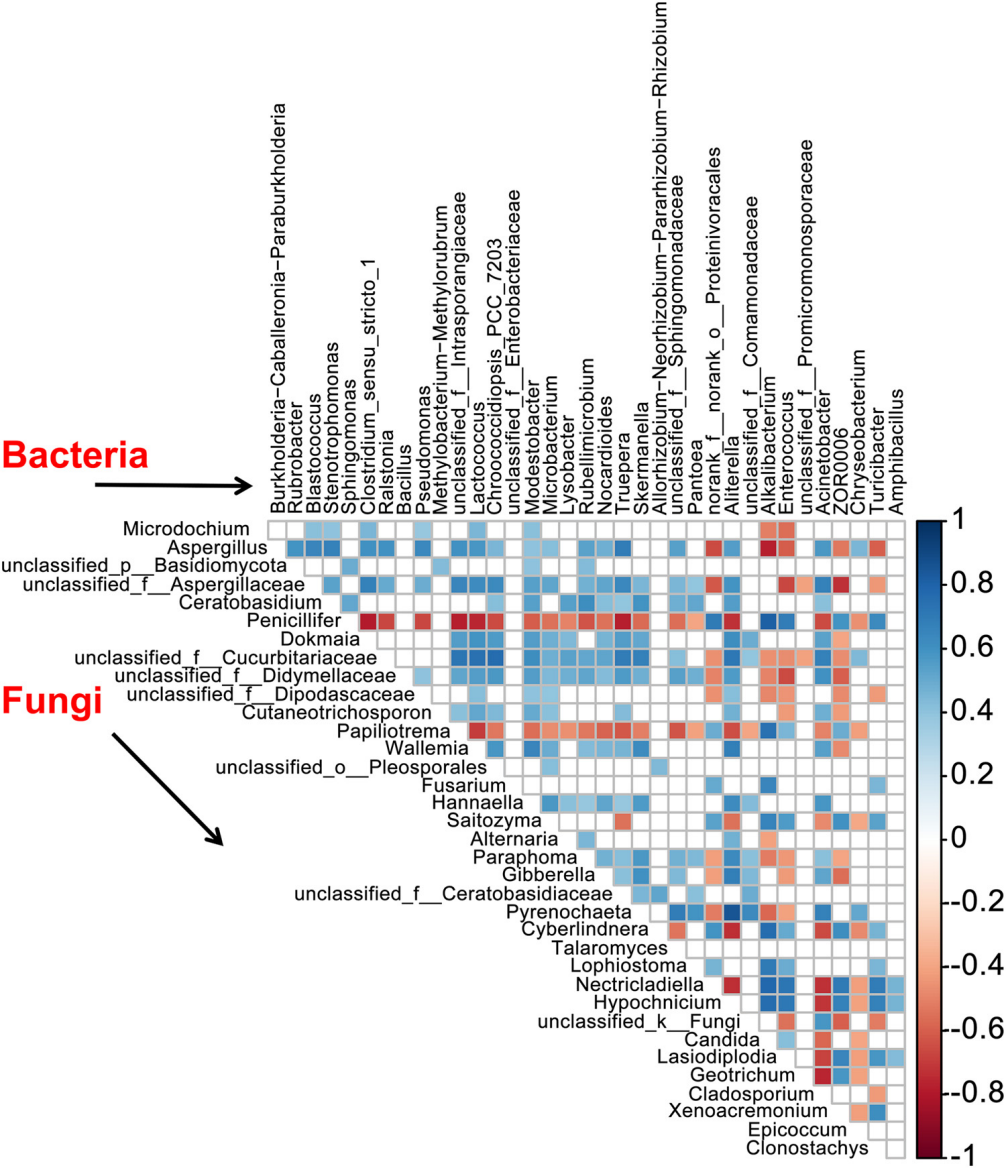
Spearman correlation listed by genus abundance (top 35). Only significant values (*P < *0.05) are shown. Orange and blue indicate significant negative and positive correlations; darker colors indicate stronger correlations.

We next assessed the Spearman’s correlation between the fungal and bacterial microbiota at the genus level. Comparison of relative abundances revealed both positive and negative correlations between the fungal and bacterial genera (Fig. 6): Aspergillus was negatively correlated with Alkalibacterium (*r* = −0.77) and Turicibacter (*r* = −0.59) but positively correlated with Stenotrophomonas (*r* = 0.68), Pseudomonas (*r* = 0.64), and Ralstonia (*r* = 0.61); Alkalibacterium was positively correlated with Penicillifer (*r* = 0.81), Nectricladiella (*r* = 0.79), Hypochnicium (*r* = 0.77), Cyberlindnera (*r* = 0.76), Papiliotrema (*r* = 0.74), Lophiostoma (*r* = 0.71), and Saitozyma (*r* = 0.67); Amphibacillus was positively correlated with Hypochnicium (*r* = 0.47), Nectricladiella (*r* = 0.46), and Lasiodiplodia (*r* = 0.43); Turicibacter was positively correlated with Nectricladiella (*r* = 0.70) and Hypochnicium (*r* = 0.68); Enterococcus was positively correlated with Nectricladiella (*r* = 0.73) and Hypochnicium (*r* = 0.72) but negatively correlated with unclassified_f__Aspergillaceae (*r* = −0.66) and unclassified_f__Didymellaceae (*r* = −0.65); and Penicillifer was negatively correlated with Pseudomonas (*r* = −0.66) and Ralstonia (*r* = −0.67).

## DISCUSSION

In the current study, the characteristic transition of chemical parameter and microbial composition during the indigo fermentation process demonstrated that the unstable nature of traditional fermentation methods could be overcome by the addition of specific plant species that generated the desired pH, redox potential, and microbiota in vat. Previous studies have indicated that appropriate pretreatment during the preparation of indigo fermentation fluids is critical for producing the desired microbial communities (3, 4). However, local craftspeople prepare fermentation vats based on personal experience and intuition, and this frequently leads to differences in microbial communities and quality between distinct batches of indigo fermentation broths. The local craftsmen occasionally fail to initiate indigo reduction through natural fermentation in a single step and thus must constantly adjust the fermentation conditions according to their specialized knowledge in order to improve the fermentation process. In this study, indigo reduction did not occur until the 27th day after the initiation of fermentation.

During the first 6 days of fermentation, we observed considerable bacterial diversity, potentially due to inappropriate pretreatment. High bacterial diversity can readily spoil the fermentation broth, whereas low bacterial diversity contributes to indigo reduction (8). Upon addition of Chinese liquor (high alcohol concentration), bacterial diversity was substantially lower than that before the addition, and Pseudomonas, Stenotrophomonas, and Bacillaceae family members propagated rapidly. Previous work has shown that the higher the alcohol concentration of the Chinese liquor added, the greater the inhibition of microbial growth (25). Thus, Chinese liquor is hypothesized to decrease the bacterial diversity, but the underlying mechanisms require further clarification.

Although adding Chinese liquor can reduce the abundance of certain microorganisms, Pseudomonas, Stenotrophomonas, and Bacillaceae family members were found to increase sharply. Previous work has indicated that the early stage of fermentation is characterized by the appearance of aerobic and facultative anaerobic Bacillaceae that consume oxygen on the 2nd day (9). However, the diversity of Gram-negative bacteria was higher than that of Gram-positive bacteria in this study. The Gram-negative bacteria Pseudomonas (26) and Alcaligenaceae have been reported as spoilage-associated bacteria during the fermentation process (8). Therefore, we conclude that Pseudomonas and Stenotrophomonas might negatively affect fermentation. Moreover, Gram-negative bacteria have been reported to antagonize facultative anaerobic and aerotolerant Bacillaceae (24). Clostridium_sensu_stricto_1 might contribute to the increased proportion of Alkalibacterium (including indigo-reducing bacteria). Alkalibacterium, a facultative anaerobe, produces l-lactic acid and grows well under both aerobic and anaerobic conditions (4), and Alkalibacterium might affect the growth of other microorganisms similar to what has been observed with other acid-producing bacteria reported previously (27–30). This could explain the decrease in Pseudomonas accompanying the appearance of Alkalibacterium. Our results also identified pH and ORP as critical factors in indigo fermentation. A long period of high pH (>11) has been reported to be detrimental to bacteria that favor indigo reduction (9), and an ORP of −600 mV is required for indigo reduction in industrial practices (31). Consequently, the pH and ORP values measured here from days 7 to 18 are not favored by this natural system. Oxygen consumption by aerobic bacteria such as Bacillaceae and the extracellular reduction by certain microorganisms have been shown to lead to ORP reduction, whereas by-products of anaerobic metabolism have been shown to cause pH change (4). However, in this study, Bacillaceae family members and Alkalibacterium were unable to reduce the ORP and pH to the desired level.

Notably, our findings suggest that the use of a specific plant mixture to adjust the fermentation process plays a crucial role in inducing indigo reduction. Although the initiation of indigo reduction varies considerably among distinct fermentation methods, the transitional changes in the microbiota necessary to initiate indigo reduction have previously been shown to require (i) a rapid decline in redox potential, (ii) lower abundance of Gram-negative than Gram-positive bacteria (24), (iii) a marked decrease in the abundance of aerobic microorganisms, (iv) dominance of aerotolerant and strict anaerobes, and (v) an overall decrease in bacterial diversity (3, 4, 9, 32). The transitional changes required to initiate the indigo reduction triggered by the addition of plant mixtures in this study are almost identical to those reported previously. Our results showed that the addition of specific plant species could not only cause an abrupt decrease in the pH and ORP but could also remove unfavorable microorganisms (such as Gram-negative or aerobic bacteria) and amplify favorable bacteria (such as aerotolerant or obligate anaerobes). The ORP value detected at the onset of indigo reduction agreed with that measured with the use of Japanese methods (5, 8), although the pH was higher and the temperature was lower than in previously reported methods (4, 6). The salt composition of plant (or wood) ash extracts could select the suitable alkalophiles during indigo fermentation, and the potassium content of straw ash is reported to be 3-fold higher than that in bark/wood fuels (33). Thus, the application of straw ash might also affect the bacterial community composition. Amphibacillus has been reported to be an indigo-reducing taxon and is frequently detected in various indigo fermentation fluids (3–5, 7). Amphibacillus appeared after the plant mixture was added, and this is presumably because Amphibacillus can decompose plant-derived macromolecules that cannot be readily decomposed by common bacteria (9). Erysipelotrichaceae are aerobic or facultatively anaerobic bacteria and have also been isolated from the fermentation broth used in Japanese methods, and these bacteria can reduce indigo (32). Enterococcus is chemo-organotrophic facultative anaerobic bacteria that produces lactic acid as the end product (34). Some Enterococcus species were first found to be provided with indigo-reducing activities (35). The appearance of the obligate anaerobe Turicibacter (36) might result from the decrease in the redox potential, but the function of Turicibacter in indigo fermentation remains to be characterized. These results suggest that previously unidentified indigo-reducing bacteria could exist in our traditional preparation method.

Clarifying the mechanism of action of the specific plant species that were selected by the local people for indigo fermentation is of considerable interest. We hypothesize that the addition of the plant mixture is crucial because this provides not only plant-associated microorganisms but also phytochemicals that act as reducing agents or electron mediators/donors for indigo reduction. Plant materials were demonstrated to contribute their attached microorganisms to the indigo fermentation fluid (24), and the endophytes of Reynoutria japonica were shown to be abundant and unique (37, 38). R. japonica was also found to contain quinone-based compounds (39); we suggest that these compounds play a key role in the dyeing process as electron mediators (40, 41) that can accelerate electron transfer. The decrease of oxygen-metabolizing or facultative anaerobic bacteria has also been reported to be related to electron mediators such as flavins or quinones (9). The chemical components of Neanotis hirsuta have not been investigated to date, but this species belongs to Rubiaceae; because diverse quinone-based compounds have been isolated from members of the Rubiaceae family (42), we assume that N. hirsuta also contains quinone-based compounds. Reducing sugars can be used as reducing agents in the indigo dyeing process (43, 44), and thus ripe fruits of Melastoma dodecandrum are added to the indigo dye vat, potentially for this reason. Flavonoid compounds have been reported to present the structural characteristics of electron donors (45); therefore, the flavonoids present in M. dodecandrum (46) might exhibit certain reducing capacities and transfer electrons to the indigo particles through an as-yet-unknown mechanism. Furthermore, a previous study (32) revealed that flavonoids exhibit strong antioxidant activity and reducing ability, which also help to reduce the content of dissolved oxygen. However, the hypotheses we have proposed regarding the addition of plants to dye vats warrant further verification in the future.

Anaerobic fungi can readily decompose crude plant biomass (47), and haloalkaliphilic fungi can produce organic acids and macromolecules (such as cellulose-degrading enzymes) (48). Our results indicated that the fungal diversity has remained in transition despite the relatively stable bacterial diversity in the proper indigo fermentation process. Anaerobic conditions were reported to favor the growth of Aspergillaceae (49), but Aspergillaceae disappeared after the plant mixture was added, and most of the bacteria that favored indigo fermentation were negatively correlated with Aspergillaceae. Moreover, Cyberlindnera was detected only after the addition of the plant mixture and was positively correlated with indigo-reducing bacteria. In the proper fermentation process, the fungal diversity has remained in transition while the bacterial diversity is relatively stable, which cannot be readily explained currently; further investigation is required into the mechanisms of action and functions of fungi during the indigo fermentation process.

### Conclusion.

Briefly, the microbial communities of the traditional indigo fermentation in China varied markedly from those reported previously, which could be attributed to the differences in the recipes or indigo dye types used for the fermentation processes, as well as to the disparities in the process parameters for characterizing the dye vats. Our results showed that the addition of specific plant species might yield the desired microbial communities and appropriate fermentation conditions; however, further studies are required to determine the functional mechanism of the plant mixture in the indigo fermentation process. Moreover, pH, ORP, and temperature were found to be critical for a stable microbial community. Although the bacterial diversity is relatively stable, the fungal diversity has remained in transition, and the mechanisms of action and functions of fungi in the indigo fermentation process remain unknown. Alkalibacterium and Amphibacillus were found to be predominant in this study, and the obligate anaerobe Turicibacter, the facultative anaerobe Enterococcus, and *ZOR0006* were identified to be specific to this traditional method. Lastly, we suggest that previously unreported indigo-reducing bacteria could also exist in our traditional dye preparation method. We expect this study to offer an environmentally friendly and economically viable alternative for industrial indigo reduction.

## MATERIALS AND METHODS

### Traditional indigo fermentation.

**FIG 7:**
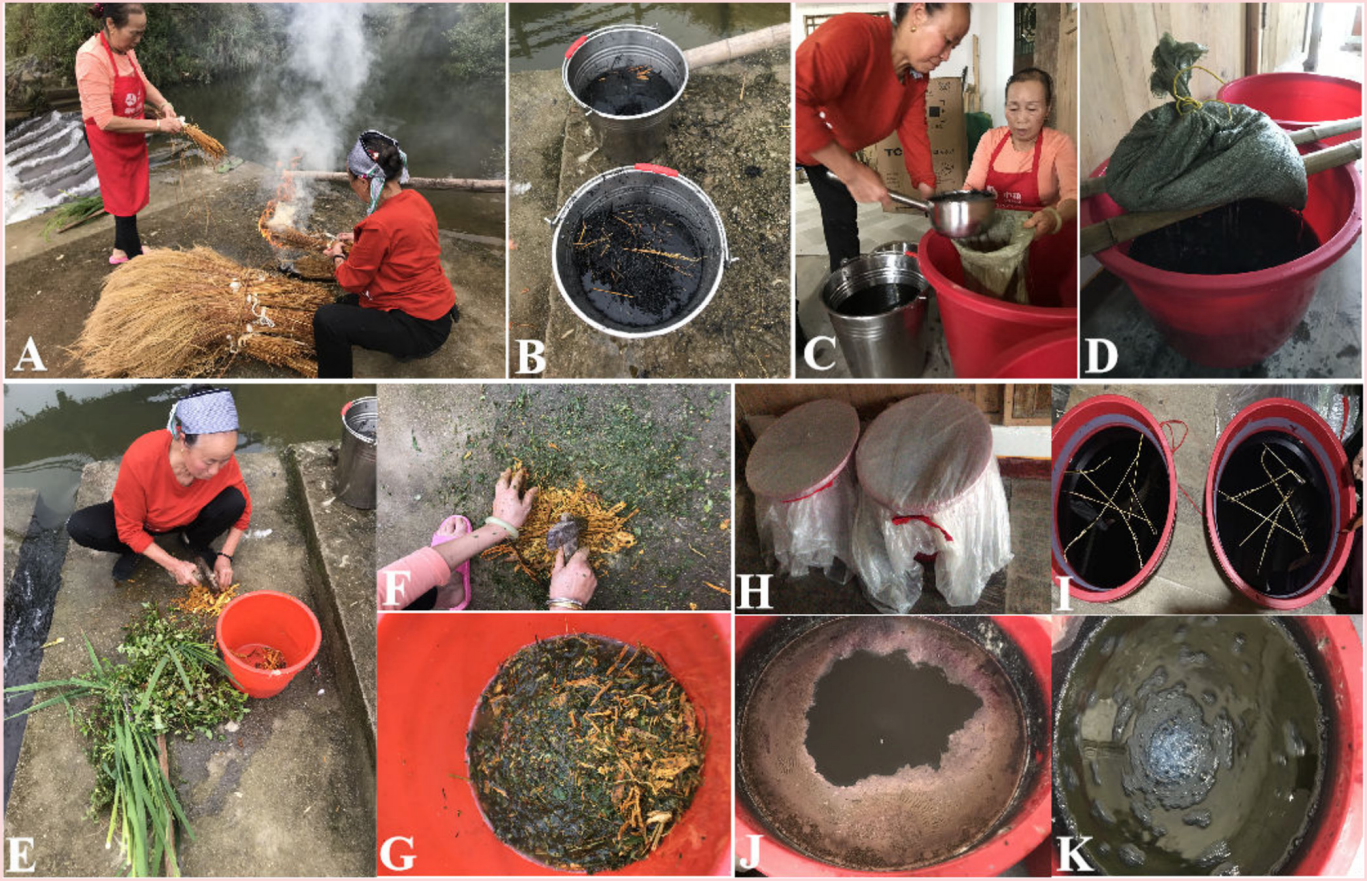
Traditional indigo vat dyeing processes used by Dong people in Hunan, China. (A) Burning sticky rice straw for obtaining alkaline ash. (B) Soaking sticky rice ash in buckets of water to produce “ash water.” (C) Filtering water through a sack filled with ash from sticky rice straw. (D) Settling the sack on a dyeing vat, which is later used to prepare a high-pH dye vat. (E) Several plant species are selected for fermentation in the traditional dyeing vat. (F) Crushing the selected plant species by using a hammer. (G) The crushed plant species to be added are soaked in the tap water. (H) The traditional dyeing vat is wrapped with plastic cloth to keep it warm. (I) The rice straw is folded up and placed on the surface of the dyeing liquid to protect the dye vat. (J) Indigo fermentation fluid is characterized by the formation of a thin film with a metallic luster on the surface of the liquid after the initial reduction. (K) The dyeing liquor in a reduced state.

The Dong communities in Gaobu Village, Tongdao County, Huaihua City, Hunan Province, China, use the following traditional indigo fermentation procedure: (i) Ash lye is produced by immersing 8.75 kg of sticky rice straw ash in 49 liters of tap water and then filtering this through a sack (Fig. 7A to D). (ii) The specific plant species (~1.5 kg), including the roots of R. japonica Houtt., fresh leaves of Oryza sativa L., and entire plants of M. dodecandrum Lour. and N. hirsuta (L.f.) W.H.Lewis, are crushed and drenched in 1.665 liters of tap water (Fig. 7E to G). (iii) The dyeing vat is prepared by adding 2.5 kg of indigo paste (indigo extracted from S. cusia and P. tinctorium), 0.5 kg of rice wine (made by local people), 49 liters of ash lye, and the aforementioned plant immersion liquid (Fig. 7H). The fermentation vat is stirred with a bar twice daily. The initial indigo reduction typically requires ~7 to 14 days during traditional indigo fermentation. However, successful fermentation relies on skilled craftspeople using their experience and intuition to adjust the process when the fermentation conditions are not suitable. In this study, the craftspeople added 500 mL of Chinese liquor (high alcohol concentration) to the fermentation fluid on the 6th day because the fluid was malodorous. Subsequently, 3 kg of the aforementioned specific plant immersion liquid was dispersed into the fermentation fluid on the 18th day, because visual inspection by the craftsmen indicated that the fermentation liquor was on the verge of deteriorating. Here, 27 days were required to optimize the indigo fermentation process in autumn (from 21 September 2020 to 17 October 2020) (Fig. 7J and K), and this was verified by dyeing cloth during our field research (Fig. S5).

### Sample collection.

With the traditional indigo dyeing vat used here, ~27 days were required for fermentation until the dye liquid turned yellow-green in color; this liquid could be used to dye cloth after the production was confirmed by the local people. In this study, we collected 26 indigo fermentation samples from the dyeing vat for sequencing. The pH, redox potential (ORP), and temperature values were measured once daily, after agitating the broth, by using an electrode sensor (InPro 3250i, Mettler-Toledo Co., Switzerland) and a potentiometer (M300, Mettler-Toledo Co., Switzerland).

### DNA extraction and PCR amplification.

After agitating the broth, the fermentation fluid, including the precipitated debris, was collected from the dyeing vat; 26 samples were collected from the 2nd day to the 27th day (the initial day of reduction) and stored at −80°C for microbiota analysis. Aliquots of the obtained indigo fermentation fluid samples were centrifuged at 15,000 × *g* for 10 min, and the supernatants were discarded. To isolate total microbial DNA, the cell pellets were extracted using a DNeasy PowerSoil kit (MoBio Laboratories, Inc., Carlsbad, CA), according to the manufacturer’s instructions. In the case of fungi, ITS region 1 (ITS1) sequences were amplified using the primers ITS1F (5′-CTTGGTCATTTAGAGGAAGTAA-3′) and ITS2R (5′-GCTGCGTTCTTCATCGATGC-3′); for bacteria, the variable region including V3 to V4 sequences of the 16S rRNA gene were amplified using forward primer 341F (5′-CCTAYGGGRBGCASCAG-3′) and reverse primer 806R (5′-GGACTACNNGGGTATCTAAT-3′). The 16S rRNA gene and ITS region were PCR-amplified under the following conditions: initial denaturation at 95°C for 3 min, followed by 29 and 35 cycles of 30 s at 95°C, 30 s at 55°C, and 45 s at 72°C, and then a single extension at 72°C for 10 min; the cycle was halted at 10°C.

### Sequencing.

For each sample, purified amplicons were pooled in equimolar concentrations and analyzed by means of paired-end sequencing (2 × 300) performed on an Illumina MiSeq platform (Illumina, San Diego, CA), according to the standard protocols of Majorbio Bio-Pharm Technology Co. Ltd. (Shanghai, China). After demultiplexing, the resulting sequences were quality filtered using fastp version 0.19.6 and merged using FLASH version 1.2.7. Next, the high-quality sequencing reads obtained were denoised by using the DADA2 plugin in the QIIME2 version 2020.2 pipeline with recommended parameters; this yields single-nucleotide resolution based on error profiles within samples. The DADA2-denoised sequences are typically called amplicon sequence variants (ASVs). For 16S rRNA gene sequencing, taxonomic analysis was done using a naive Bayes classifier trained on the SILVA 16S rRNA database (version 138), and for ITS sequencing, UNITE 8.0 database.

### Statistical analysis.

Statistics and plotting were performed using R (version 4.1.1). Relative abundances of specific samples were computed using the “arrange” function from dplyr package and visualized using ggplot2 package (50), and the α-diversity index was calculated using the diversity function of the vegan package (51). The Bray-Curtis distances were computed for PCoA by using the “vegdist” function in vegan package and the “pcoa” function in ape package and then plotted using ggplot2 package (52). Venn diagrams were generated, using R package “venn,” to visualize the shared and unique genera before and after the specific plant species were added to the dye vat. For CCA, the sorting analysis was performed using the “cca” function from vegan package. The *r*^2^ and *P* values of the impact of each environmental factor on sample distribution were calculated using the “envfit” function in vegan package, after which the significant environmental factors were screened for by using CCA (53). The results of linear-regression analysis were visualized using the “ggscatter” function of the ggpubr package (50). Correlation analyses were performed on the 35 most abundant genera of bacteria and fungi. Association both between and within the fungal and bacterial species at the genus level was calculated using the built-in Spearman correlation analysis in the “rcorr” function of the Hmisc package (54). Furthermore, the correlation matrix of genera was visualized using the “corrplot” function of corrplot package (55).

### Data availability.

The sequence data associated with this project have been deposited in the NCBI Sequence Read Archive (SRA) database under the accession number PRJNA835230.

## References

[1] Splitstoser JC, Dillehay TD, Wouters J, Claro A. 2016. Early pre-Hispanic use of indigo blue in Peru. Sci Adv 2:1–5. doi:10.1126/sciadv.1501623. PMC5023320 27652337

[2] Cardon D. 2007. Natural Dyes: Sources, Tradition, Technology and Science. Archetype Books, London, England.

[3] Tu Z, de Fátima Silva Lopes H, Hirota K, Yumoto I. 2019. Analysis of the microbiota involved in the early changes associated with indigo reduction in the natural fermentation of indigo. World J Microbiol Biotechnol 35:1–9. doi:10.1007/s11274-019-2699-5. 31346774

[4] Aino K, Hirota K, Okamoto T, Tu Z, Matsuyama H, Yumoto I. 2018. Microbial communities associated with indigo fermentation that thrive in anaerobic alkaline environments. Front Microbiol 9:2196. doi:10.3389/fmicb.2018.02196. 30279681 PMC6153312

[5] Aino K, Narihiro T, Minamida K, Kamagata Y, Yoshimune K, Yumoto I. 2010. Bacterial community characterization and dynamics of indigo fermentation. FEMS Microbiol Ecol 74:174–183. doi:10.1111/j.1574-6941.2010.00946.x. 20695891

[6] Milanovic V, Osimani A, Taccari M, Garofalo C, Butta A, Clementi F, Aquilanti L. 2017. Insight into the bacterial diversity of fermentation woad dye vats as revealed by PCR-DGGE and pyrosequencing. J Ind Microbiol Biotechnol 44:997–1004. doi:10.1007/s10295-017-1921-4. 28246965

[7] Okamoto T, Aino K, Narihiro T, Matsuyama H, Yumoto I. 2017. Analysis of microbiota involved in the aged natural fermentation of indigo. World J Microbiol Biotechnol 33:1–10. doi:10.1007/s11274-017-2238-1. 28285451

[8] Tu Z, de Fátima Silva Lopes H, Igarashi K, Yumoto I. 2019. Characterization of the microbiota in long- and short-term natural indigo fermentation. J Ind Microbiol Biotechnol 46:1657–1667. doi:10.1007/s10295-019-02223-0. 31432338

[9] Tu Z, Lopes H, de FS, Narihiro T, Yumoto I. 2021. The mechanism underlying of long-term stable indigo reduction state in indigo fermentation using sukumo (composted *Polygonum tinctorium* leaves). Front Microbiol 12:698674. doi:10.3389/fmicb.2021.698674. 34367099 PMC8342947

[10] Hirota K, Aino K, Nodasaka Y, Morita N, Yumoto I. 2013. *Amphibacillus indicireducens* sp. nov., an alkaliphile that reduces an indigo dye. Int J Syst Evol Microbiol 63:464–469. doi:10.1099/ijs.0.037622-0. 22493173

[11] Hirota K, Aino K, Nodasaka Y, Yumoto I. 2013. *Oceanobacillus indicireducens* sp. nov., a facultative alkaliphile that reduces an indigo dye. Int J Syst Evol Microbiol 63:1437–1442. doi:10.1099/ijs.0.034579-0. 22843722

[12] Hirota K, Aino K, Yumoto I. 2013. *Amphibacillus iburiensis* sp. nov., an alkaliphile that reduces an indigo dye. Int J Syst Evol Microbiol 63:4303–4308. doi:10.1099/ijs.0.048009-0. 23832971

[13] Hirota K, Aino K, Yumoto I. 2016. *Fermentibacillus polygoni* gen. nov., sp. nov., an alkaliphile that reduces indigo dye. Int J Syst Evol Microbiol 66:2247–2253. doi:10.1099/ijsem.0.001015. 26971318

[14] Hirota K, Nishita M, Matsuyama H, Yumoto I. 2017. *Paralkalibacillus indicireducens* gen., nov., sp. nov., an indigo-reducing obligate alkaliphile isolated from indigo fermentation liquor used for dyeing. Int J Syst Evol Microbiol 67:4050–4056. doi:10.1099/ijsem.0.002248. 28905696

[15] Hirota K, Nishita M, Tu Z, Matsuyama H, Yumoto I. 2018. *Bacillus fermenti* sp. nov., an indigo-reducing obligate alkaliphile isolated from indigo fermentation liquor for dyeing. Int J Syst Evol Microbiol 68:1123–1129. doi:10.1099/ijsem.0.002636. 29458563

[16] Hirota K, Okamoto T, Matsuyama H, Yumoto I. 2016. *Polygonibacillus indicireducens* gen. nov., sp. nov., an indigo-reducing and obligate alkaliphile isolated from indigo fermentation liquor for dyeing. Int J Syst Evol Microbiol 66:4650–4656. doi:10.1099/ijsem.0.001405. 27503611

[17] Nakajima K, Hirota K, Nodasaka Y, Yumoto I. 2005. *Alkalibacterium iburiense* sp. nov., an obligate alkaliphile that reduces an indigo dye. Int J Syst Evol Microbiol 55:1525–1530. doi:10.1099/ijs.0.63487-0. 16014476

[18] Padden AN, John P, Collins MD, Hutson R, Hall AR. 2000. Indigo-reducing *Clostridium isatidis* isolated from a variety of sources, including a 10th-century Viking dye vat. J Archaeol Sci 27:953–956. doi:10.1006/jasc.1999.0524.

[19] Park S, Lee JH, Cho YJ, Chun J, Hur HG. 2013. Draft genome sequence of *Pseudomonas* sp. strain G5, isolated from a traditional indigo fermentation dye vat. J Korean Soc Appl Biol Chem 56:339–341. doi:10.1007/s13765-013-3033-9.

[20] Spanka R, Fritze D. 1993. *Bacillus cohnii* sp. nov., a new, obligately alkaliphilic, oval-spore-forming *Bacillus* species with ornithine and aspartic acid instead of diaminopimelic acid in the cell wall. Int J Syst Evol Microbiol 43:150–156. 10.1099/00207713-43-1-150 8323866

[21] Tu Z, Lopes HdFS, Yumoto I. 2022. *Fundicoccus fermenti* sp. nov., an indigo-reducing facultative anaerobic alkaliphile isolated from indigo fermentation liquor used for dyeing. Int J Syst Evol Microbiol 72:1–9. doi:10.1099/ijsem.0.005239. 35156919

[22] Yumoto I, Hirota K, Nodasaka Y, Tokiwa Y, Nakajima K. 2008. *Alkalibacterium indicireducens* sp. nov., an obligate alkaliphile that reduces indigo dye. Int J Syst Evol Microbiol 58:901–905. doi:10.1099/ijs.0.64995-0. 18398191

[23] Yumoto I, Hirota K, Nodasaka Y, Yokota Y, Hoshino T, Nakajima K. 2004. *Alkalibacterium psychrotolerans* sp. nov., a psychrotolerant obligate alkaliphile that reduces an indigo dye. Int J Syst Evol Microbiol 54:2379–2383. doi:10.1099/ijs.0.63130-0. 15545487

[24] Lopes HdFS, Tu Z, Sumi H, Yumoto I. 2021. Analysis of bacterial flora of indigo fermentation fluids utilizing composted indigo leaves (sukumo) and indigo extracted from plants (Ryukyu-ai and Indian indigo). J Biosci Bioeng 132:279–286. doi:10.1016/j.jbiosc.2021.05.004. 34127379

[25] Hu D, Xu Y, Yu D, Xia W, Jiang Q. 2020. The impacts of salt with Chinese liquor on the inhibition of microbial spoilage and quality attributes of grass carp (*Ctenopharyngodon idellus*) fillets stored at 4° C. J Food Process Preserv 44:e14817. doi:10.1111/jfpp.14817.

[26] Wang X, Du H, Zhang Y, Xu Y. 2018. Environmental microbiota drives microbial succession and metabolic profiles during Chinese liquor fermentation. Appl Environ Microbiol 84:e02369-17. doi:10.1128/AEM.02369-17. 29196296 PMC5795089

[27] Antunes M, Palma M, Sá-Correia I. 2018. Transcriptional profiling of *Zygosaccharomyces bailii* early response to acetic acid or copper stress mediated by ZbHaa1. Sci Rep 8:1–14. doi:10.1038/s41598-018-32266-9. 30237501 PMC6147978

[28] Ma S, Luo H, Zhao D, Qiao Z, Zheng J, An M, Huang D. 2022. Environmental factors and interactions among microorganisms drive microbial community succession during fermentation of Nongxiangxing daqu. Bioresour Technol 345:126549. doi:10.1016/j.biortech.2021.126549. 34902488

[29] Nie Z, Zheng Y, Wang M, Han Y, Wang Y, Luo J, Niu D. 2013. Exploring microbial succession and diversity during solid-state fermentation of Tianjin duliu mature vinegar. Bioresour Technol 148:325–333. doi:10.1016/j.biortech.2013.08.152. 24055975

[30] Wu C, Huang J, Zhou R. 2017. Genomics of lactic acid bacteria: current status and potential applications. Crit Rev Microbiol 43:393–404. doi:10.1080/1040841X.2016.1179623. 28502225

[31] Bechtold T, Burtscher E, Amann A, Bobleter O. 1993. Alkali-stable iron complexes as mediators for the electrochemical reduction of dispersed organic dyestuffs. Faraday Trans 89:2451–2456. doi:10.1039/ft9938902451.

[32] Lopes HdFS, Tu Z, Sumi H, Furukawa H, Yumoto I. 2021. Indigofera tinctoria leaf powder as a promising additive to improve indigo fermentation prepared with sukumo (composted *Polygonum tinctorium* leaves). World J Microbiol Biotechnol 37:1–19. doi:10.1007/s11274-021-03142-y. 34562162

[33] Olanders B, Steenari BM. 1995. Characterization of ashes from wood and straw. Biomass Bioenergy 8:105–115. doi:10.1016/0961-9534(95)00004-Q.

[34] García-Solache M, Rice LB. 2019. The *Enterococcus*: a model of adaptability to its environment. Clin Microbiol Rev 32:e00058-18. doi:10.1128/CMR.00058-18. 30700430 PMC6431128

[35] Nakagawa K, Takeuchi M, Tada M, Matsunaga M, Kugo M, Kiyofuji S, Kikuchi M, Yomota K, Sakamoto T, Kano K, Ogawa J, Sakuradani E. 2022. Isolation and characterization of indigo-reducing bacteria and analysis of microbiota from indigo fermentation suspensions. *Bioscience*. Biosci Biotechnol Biochem 86:273–281. doi:10.1093/bbb/zbab209. 34864880

[36] Bosshard PP. 2015. Turicibacter. *In* Bergey’s Manual of Systematics of Archaea and Bacteria. Wiley, Hoboken, NJ.

[37] Yu J, Xu Q, Li Z, Liu H, Hao Z, Li H. 2021. Isolation and identification of endophytic fungi from *Polygonum cuspidatum* for polydatin transformation. Food Ferment Industries 47:21–26.

[38] Zhang Y, Zheng L, Zheng Y, Xue S, Zhang J, Huang P, Zhao Y, Hao X, He Z, Hu Z, Zhou C, Chen Q, Liu J, Wang G, Sang M, Sun X, Wang X, Xiao X, Li C. 2020. Insight into the assembly of root-associated microbiome in the medicinal plant Polygonum cuspidatum. Industrial Crops Products 145:112163. doi:10.1016/j.indcrop.2020.112163.

[39] Wang X, Hu H, Wu Z, Fan H, Wang G, Chai T, Wang H. 2021. Tissue-specific transcriptome analyses reveal candidate genes for stilbene, flavonoid and anthraquinone biosynthesis in the medicinal plant *Polygonum cuspidatum*. BMC Genomics 22:1–17. 34000984 10.1186/s12864-021-07658-3 PMC8127498

[40] Li T, Yang X, Chen Q, Song H, He Z, Yang Y. 2020. Enhanced performance of microbial fuel cells with electron mediators from anthraquinone/polyphenol-abundant herbal plants. ACS Sustainable Chem Eng 8:11263–11275. doi:10.1021/acssuschemeng.0c03058.

[41] Rau J, Knackmuss HJ, Stolz A. 2002. Effects of different quinoid redox mediators on the anaerobic reduction of azo dyes by bacteria. Environ Sci Technol 36:1497–1504. doi:10.1021/es010227. 11999057

[42] Wijnsma R, Verpoorte R. 1986. Anthraquinones in the *Rubiaceae*, p 79–149. *In* Progress in the chemistry of organic natural products. Springer, Berlin, Germany.

[43] Shin Y, Choi M, Yoo ID. 2013. Utilization of fruit by-products for organic reducing agent in indigo dyeing. Fibers Polym 14:2027–2031. doi:10.1007/s12221-013-2027-x.

[44] Shin Y, Choi M, Yoo DIL. 2014. Eco-friendly indigo reduction using bokbunja (*Rubus coreanus* Miq.) sludge. Fashion Textiles 1:1–8. doi:10.1186/s40691-014-0006-5.

[45] Zheng YZ, Deng G, Liang Q, Chen DF, Guo R, Lai RC. 2017. Antioxidant activity of quercetin and its glucosides from propolis: a theoretical study. Sci Rep 7:7543. doi:10.1038/s41598-017-08024-8. 28790397 PMC5548903

[46] Tong Y, Jiang Y, Chen X, Li X, Wang P, Jin Y, Cheng K. 2019. Extraction, enrichment, and quantification of main antioxidant aglycones of flavonoids and tannins from *Melastoma dodecandrum* Lour.: guided by UPLC-ESI-MS/MS. J Chem 2019:1–12. doi:10.1155/2019/2793058.

[47] Hooker CA, Lee KZ, Solomon KV. 2019. Leveraging anaerobic fungi for biotechnology. Curr Opin Biotechnol 59:103–110. doi:10.1016/j.copbio.2019.03.013. 31005803

[48] Wei Y, Zhang S. 2018. Abiostress resistance and cellulose degradation abilities of haloalkaliphilic fungi: applications for saline–alkaline remediation. Extremophiles 22:155–164. doi:10.1007/s00792-017-0986-3. 29290045

[49] Yang C, Sun J. 2020. Soil salinity drives the distribution patterns and ecological functions of fungi in saline-alkali land in the Yellow River Delta, China. Front Microbiol 11:594284. doi:10.3389/fmicb.2020.594284. 33424797 PMC7786015

[50] Kassambara A. 2020. ggpubr: ggplot2 based publication ready plots. R package version 0.4.0.

[51] Dixon P. 2003. vegan, a package of R functions for community ecology. J Veg Sci 14:927–930. doi:10.1111/j.1654-1103.2003.tb02228.x.

[52] Ren B, Shi X, Chi Y, Ren T, Jin X, Wang XC, Jin P. 2022. A comprehensive assessment of fungi in urban sewer biofilms: community structure, environmental factors, and symbiosis patterns. Science Total Environ 806:150728. doi:10.1016/j.scitotenv.2021.150728. 34606856

[53] González I, Déjean S, Martin PGP, Baccini A. 2008. CCA: an R package to extend canonical correlation analysis. J Stat Soft 23:1–14. doi:10.18637/jss.v023.i12.

[54] Harrell FE, Jr, Harrell MFE, Jr. 2019. Package hmisc. CRAN2018 2019:235–236.

[55] Wei T, Simko V, Levy M, Xie Y, Jin Y, Zemla J. 2017. Package corrplot. Statistician 56:e24.

