# Supplementary material for: Fermentation Blues: Analyzing the Microbiota of Traditional Indigo Vat Dyeing in Hunan, China
Source: Microbiol Spectr. 2022 Jun 16;10(4):e01663-22. doi: 10.1128/spectrum.01663-22 (PMC9430710; doi:10.1128/spectrum.01663-22)
Supplement: Supplemental file 1 — Supplemental material. Download spectrum.01663-22-s0001.pdf, PDF file, 0.4 MB [file spectrum.01663-22-s0001.pdf]

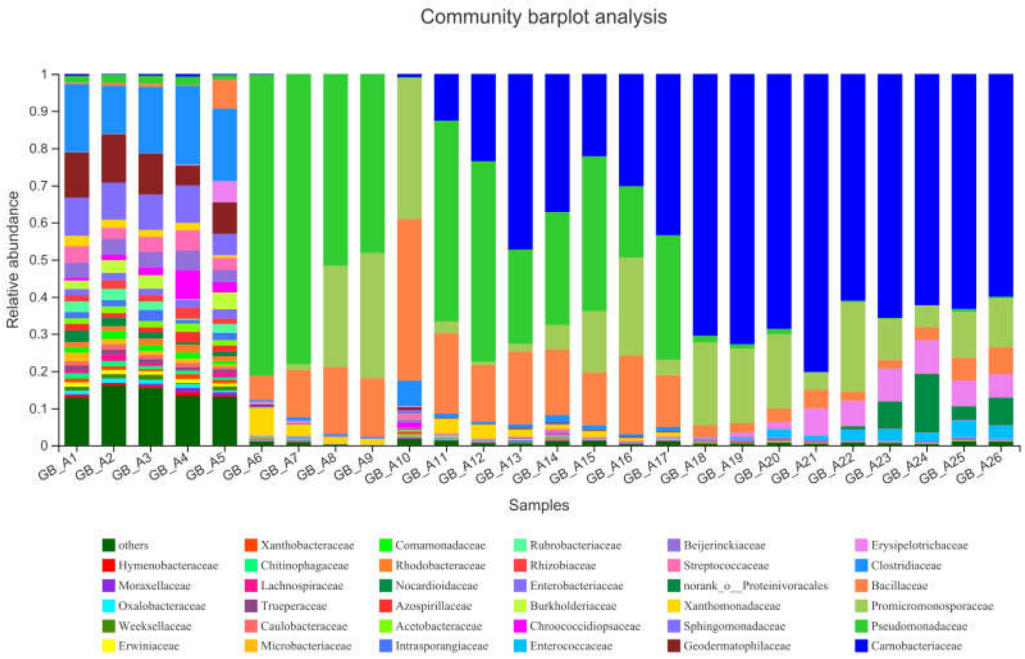

Fig. S1. Relative abundance of bacterial families in indigo fermentation fluids over time. Bacterial families detected at a relative abundance of ≤ 1% are classified as “Others”.

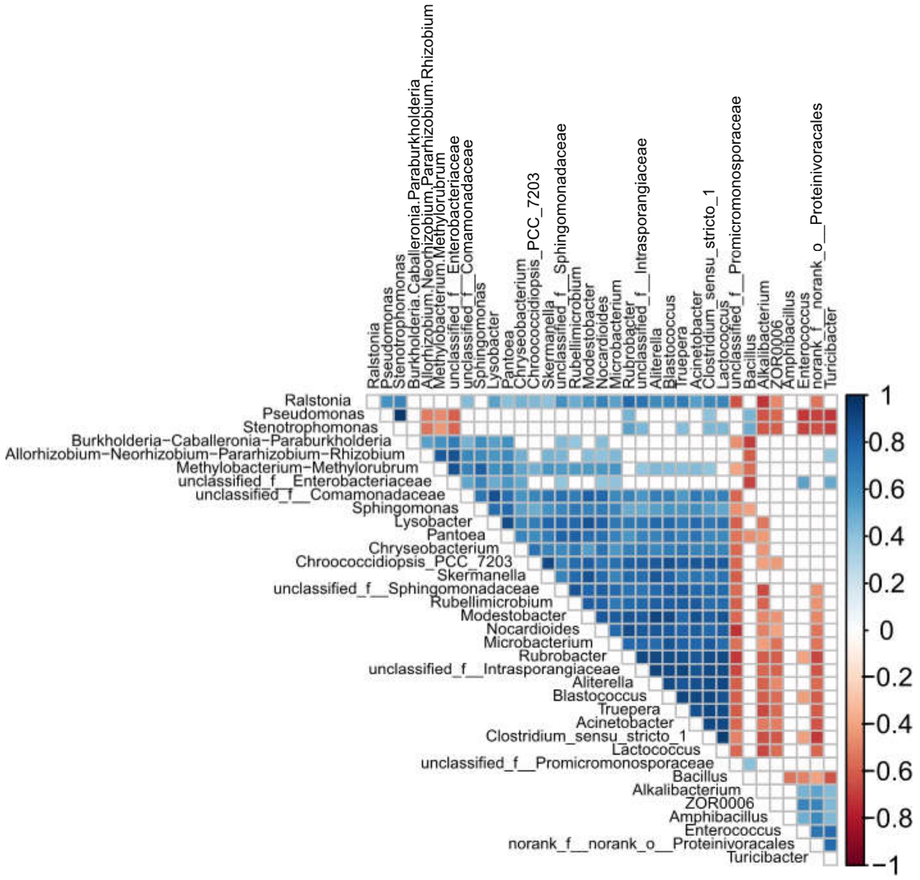

Fig. S2. Spearman correlation between bacteria in indigo fermentation samples (top 35). Only significant values (*P* < 0.05) are shown. Orange and blue: significant negative and positive correlations, respectively; darker colors: stronger correlations.

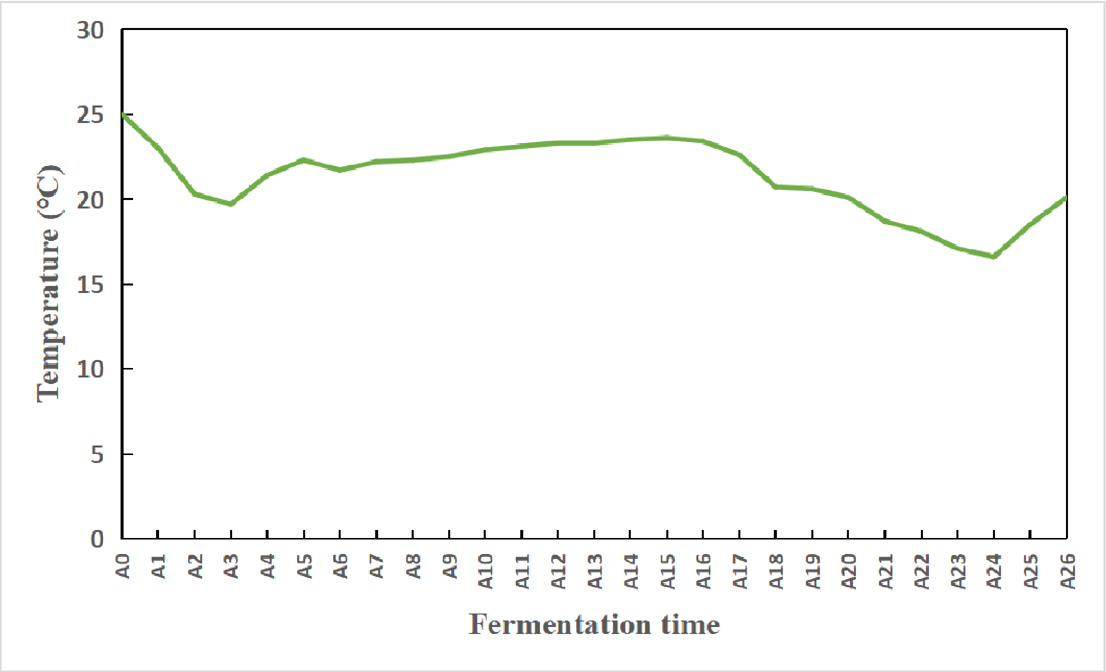

Fig. S3. The changes of temperature in the indigo fermentation process.

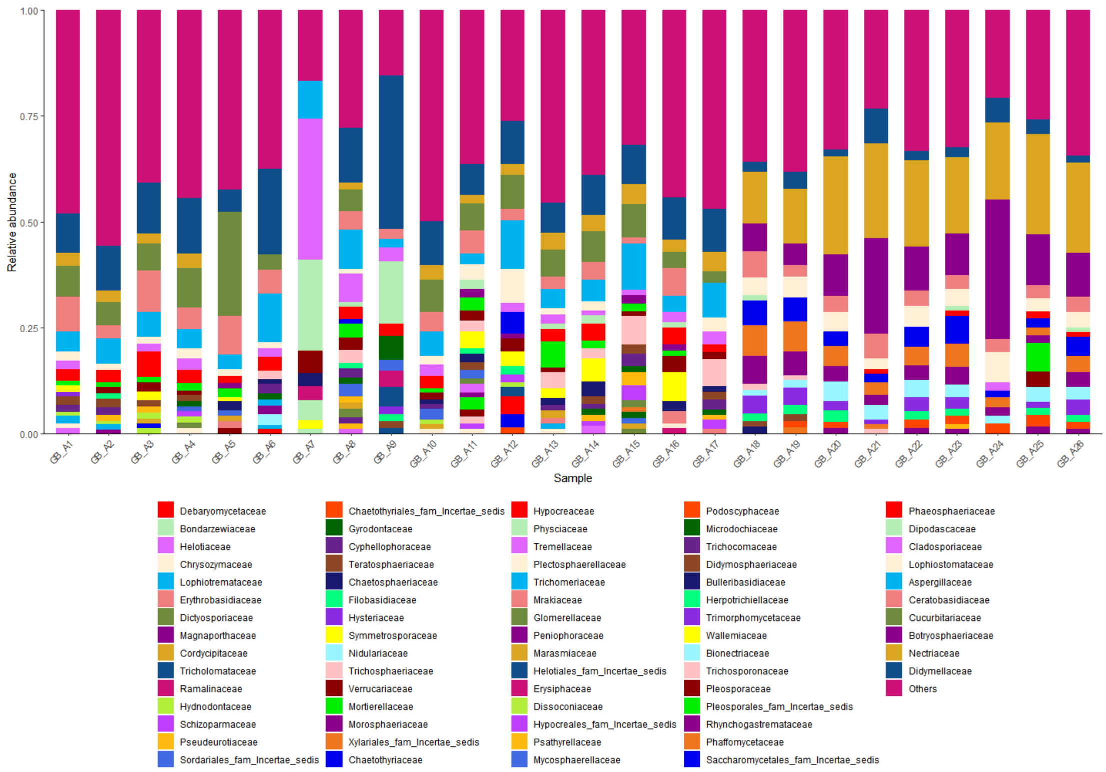

Fig. S4. Relative abundance of fungal families in indigo fermentation fluids over time. Fungal families detected at a relative abundance of ≤ 1% are classified as “Others”.

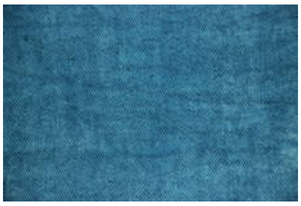

Fig. S5. The dyed cloth by local people.
